# Combined consideration of body mass index and waist circumference identifies obesity patterns associated with risk of stroke in a Chinese prospective cohort study

**DOI:** 10.1186/s12889-022-12756-2

**Published:** 2022-02-18

**Authors:** Xiangfeng Cong, Shaobo Liu, Wenjuan Wang, Jixiang Ma, Jianhong Li

**Affiliations:** 1 National Center for Chronic and Non-communicable Disease Control and Prevention, Chinese Center for Disease Control and Prevention, 100050 Beijing, China; 2 Office of Non-Communicable Diseases and Ageing Health Management, Chinese Center for Disease Control and Prevention, 102206 Beijing, China

**Keywords:** Body mass index, Waist circumference, Stroke, Prospective cohort

## Abstract

**Background:**

In China, few studies have examined the relationship between the combination of body mass index and waist circumference and the risk of stroke. Moreover, the relationship may also be different in different genders. Thus, we investigated the association between the combination of body mass index and waist circumference and the risk of stroke in Chinese.

**Methods:**

This prospective cohort study included 36 632 participants aged 18 to 90 years. Participants were recruited from 60 surveillance sites (25 urban sites and 35 rural sites) across China in 2010 China Chronic Disease Risk Factor Surveillance, and followed up in 2016-2017. Incident cases of stroke were identified through questionnaires (including the basis of clinical diagnosis, imaging tests, time of diagnosis, diagnosis unit) and Cardiovascular Event Report System. Risk factors for stroke were collected at baseline using questionnaire, physical measurements and laboratory tests. Cox proportional hazards regression models were used to generate adjusted hazard ratios and 95%CI. All analyses were duplicated by gender stratification.

**Results:**

During 6.42 ± 0.50 years of follow-up, 1 333 (597 males, 736 females) stroke events were observed among the 27 112 participants who did not have cardiovascular diseases at baseline. Compared with the general population who have normal weight or underweight with normal WC, those who have normal weight or underweight with abdominal obesity (adjusted hazard ratios 1.45, 95%CI 1.07-1.97 in males; 0.98, 95%CI 0.78-1.24 in females), overweight with abdominal obesity (1.41, 95%CI 1.14-1.75 in males; 1.33, 95%CI 1.10-1.61 in females), obesity with abdominal obesity (1.46, 95%CI 1.11-1.91 in males; 1.46, 95%CI 1.17-1.81 in females). Overweight with normal WC was found to be not statistically significant for both males and females (all *P*>0.05). Subgroup analysis found a multiplicative interaction between age and anthropometric group in females (*P* for interaction <0.05). Sensitivity analysis results did not change. In the subjects with CVD risk factors, we found a similar relationship as in the general population .

**Conclusions:**

Combined assessment of body mass index and waist circumference identifies obesity patterns associated with stroke risk.

**Supplementary Information:**

The online version contains supplementary material available at 10.1186/s12889-022-12756-2.

## Background

Stroke is a leading cause of death, disability, and a significant contributor to economic burden [1–3]. There were 5.5 million deaths due to stroke and 7 million new stroke cases worldwide in 2016 [4]. In China, stroke has become the highest burden of disease [5]. The number of stroke patients is about 13 million, and the age-standardized prevalence, incidence, and mortality rates of stroke were 11.2/1000 people, 2.5 and 1.2/1000 person-years, respectively [6, 7]. Such a heavy disease burden of stroke has been of great concern. Identifying and modifying risk factors is essential for controlling the epidemic of stroke.

Numerous studies confirmed that obesity (defined by body mass index) is an important risk factor for stroke onset and its subtypes [8–11]. Body mass index (BMI) is a common indicator of obesity in China [12]. However, this indicator (BMI) has some limitations. For instance, it cannot detect obesity levels of abdominal fat [13]. However, fat distribution may be an important factor in the development of stroke, such as the ratio of gynoid fat mass to total fat mass which is inversely associated with hypertriglyceridemia and hypertension [14, 15]. Adipose tissue constitutes a highly active endocrine organ, and adipocytes can synthesize potential anti-atherosclerotic proteins, such as adiponectin which is a fat-derived hormone that appears to play a crucial role in protecting against insulin resistance/diabetes and atherosclerosis important mechanisms involved in the pathogenesis of stroke [16–20]. It has been suggested that waist circumference (WC) can complement body mass index to assess abdominal obesity [21, 22]. The combination of BMI and WC might be better to evaluate the fat distribution. Recent studies also showed that people with normal weight abdominal obesity had a higher mortality risk and overweight without abdominal obesity had a lower mortality risk, demonstrating the importance of combining body mass index and waist circumference [23, 24].

However, studies on the relationship between the combination of body mass index and waist circumference and stroke onset are limited in China. Moreover, this relationship is not consistent in different gender, country, and region [25, 26]. Chinese people have a higher percentage of body fat than Caucasians [27]. Therefore, it is necessary to explore the relationship between the combination of body mass index and waist circumference and stroke onset in Chinese population. We used a cohort study to analyze the risk of stroke in different anthropometric groups according to BMI and WC combinations in Chinese males and females and providing epidemiological evidence for weight control to prevent stroke in China.

## Methods

### Study design and baseline survey

This study is a prospective cohort study to investigate the association between the combination of body mass index and waist circumference and the risk of stroke in Chinese. According to 2010 China Chronic Disease Risk Factor Surveillance (CCDRFS) 162 eastern, central and western surveillance sites distribution and fitting, we selected 60 (35 rural and 25 urban) surveillance sites to cover a wide range of risk exposures. And a total of 36 632 participants aged 18 to 90 years were included at baseline in 2010. For more details of the CCDRFS have been reported previously [28, 29]. Our study passed the review of the Ethics Review Committee of the National Center for Chronic and Non-communicable Disease Control and Prevention, Chinese Center for Disease Control and Prevention (approval number: 201524B). All methods were performed in accordance with the Declaration of Helsinki. All survey respondents have signed informed consent.

The 2010 baseline survey consisted of questionnaires (household and individual) through face-to-face survey, physical measurements, and laboratory tests. The questionnaire included basic personal information, lifestyle, and health status. Physical measurements included height measurement, weight measurement, waist measurement and blood pressure. Measurements were carried out by trained and qualified investigators using standard methods. Laboratory tests included fasting blood glucose, oral glucose tolerance test-2 h (OGTT-2 h), total cholesterol (TC), low-density lipoprotein cholesterol (LDL-C), high-density lipoprotein cholesterol (HDL-C), triglycerides (TG). Detailed information on the collection, preservation, processing of the blood samples has been described previously [28, 30, 31].

### Definition and grouping of the indicators

(1) Anthropometric group: subjects were grouped according to BMI (normal weight or underweight: <24 kg/m^2^, overweight: 24-27.9 kg/m^2^ and obesity: ≥28 kg/m^2^) and WC (normal WC: men’s WC <85 cm, women’s <80 cm; and abdominal obesity: men’s WC ≥85 cm, women’s ≥80 cm) [32]. Subjects were divided into five anthropometric groups: ①normal weight or underweight/normal WC, ② overweight/normal WC, ③ normal weight or underweight/abdominal obesity, ④overweight/abdominal obesity, and ⑤ obesity/abdominal obesity. Obesity is not further divided by waist circumference into obesity normal WC and obesity abdominal obesity, both considered obesity abdominal obesity. Because most obese individuals had a waist circumference above the cut-off point for abdominal obesity, only 166 of our respondents were obese with normal WC. (2) The subjects with CVD risk factors was defined as people with diabetes mellitus and (or) hypertension and (or) low HDL-C at baseline.

### Follow-up and outcome measures

A follow-up survey of the population at the 60 surveillance sites was conducted in 2016-2017. The survey was conducted in the form of a door-to-door interview. The questionnaire consisted of a household questionnaire and an individual questionnaire. For deceased subjects, the relevant information was collected through the China Death Cause Registration Reporting System [33, 34]. Respondents who moved away from the survey site were interviewed by telephone about cardiovascular diseases and completed a questionnaire.

For identifying stroke cases, diagnostic information was collected through questionnaires (including the basis of clinical diagnosis, imaging tests, time of diagnosis, diagnosis unit), and medical records and images were also reviewed. If the survey site has a Cardiovascular Event Reporting System, the questionnaire data was also be checked through the system. According to the *International Classification of Diseases, 10th Revision* (ICD-10), the stroke outcomes in our study were subarachnoid haemorrhage (I60), intracerebral haemorrhage (I61), and cerebral infarction (I63). For analysis of incident stroke in our study, only the first stroke event was counted. Overall, 27 762 people were followed up, including 814 who died and 238 died of stroke. All follow-up subjects have signed informed consent.

### Statistical analysis

A total of 27 112 participants were included in the analysis (Flowchart of participant inclusion see Figure S1 in the Additional file 1). The follow-up time was defined as the time interval between the date of the baseline interview and the date of stroke diagnosis, death of any cause, or of the follow-up survey, We analyzed males and females subjects separately, for each of the baseline anthropometric groups. We used the Cox proportional hazards model to calculate adjusted hazard ratios for the relationship between anthropometric group and stroke. The proportional hazards assumption for all variables was confirmed using the Schoenfeld residuals method. We also used the floating absolute risk (FAR) method to calculate the 95% confidence intervals for the hazard ratios. The method can be compared between different exposure categories, including non-reference categories[35]. Multiplicative interaction tests used likelihood ratio tests to compare statistical significance between models with and without interaction items. Sensitivity analyses were performed by excluding those who died and those diagnosed with stroke within the first year and repeating the Cox proportional hazards model above.

Cox models were adjusted by considering confounder factors including geographic region (eastern, central, western), age, educational level (no formal education, primary school, middle school, high school and above), marital status (single, married/cohabiting, separated/divorced/widowed), location (urban or rural), occupational (agriculture/forestry/animal-husbandry/fishing workers, business service personnel, professional/managerial personnel, retired personnel, unemployed/others), smoking (never, previous, current), current drinking (yes or no), physical activity, self-rated health (excellent, good, fair, poor), consumption of fresh vegetables (<4 days/week, 4-6 days/week, daily), consumption of fresh fruit(never/rarely, 1-2 days/week, 3-6 days/week, daily), and insufficient intake of vegetables and fruit(yes or no).

SAS software, version 9.4 (SAS Institute.) was used to for the data analysis. The statistical significance level was set at *P *< 0.05, and all *P* values were given for 2-sided tests.

## Results

### Baseline Characteristics

According to the anthropometric group, the baseline characteristics of 27 112 study population are presented in Table 1. In males, the mean BMI (standard deviation) was 23.9 (3.4) kg/m^2^, the mean WC (standard deviation) was 82.6 (10.2) cm. The prevalence of general obesity was 11.8% (95%CI: 11.2-12.3%), and abdominal obesity was 40.1% (95%CI: 39.2-41.0%) (see Figure. S2 and Figure. S3 in the Additional file 1). In females, the mean BMI (standard deviation) was 24.2 (3.6) kg/m^2^, the mean WC (standard deviation) was 79.8 (9.9) cm. The prevalence of general obesity was 14.6% (95%CI: 14.0-15.1%), and abdominal obesity was 47.9% (95%CI: 47.1-48.7%) (see Figure. S2 and Figure. S3 in the Additional file 1).

**Table 1:** Baseline Characteristic of Included Participants by BMI and WC

Male	Overall	Normal weight or underweight/ Normal WC	Overweight/ Normal WC	Normal weight or underweight/ Abdominal obesity		Overweight/ Abdominal obesity	Obesity/ Abdominal obesity
Number	12 259 (100.0)	5 984 (48.8)	1 291 (10.5)	830 (6.8)		2 713 (22.1)	1 441 (11.8)
Age, M (P25, P75), y	47 (38, 58)	48 (37, 59)	47 (38, 57)	50 (40, 60)		48 (40, 58)	46 (38, 56)*
Urban residence, n (%)	4 581 (37.4)	1 899 (31.7)	462 (35.8)	310 (37.3)		1 259 (46.4)	651 (45.2)*
High school and above, n (%)	2 910 (23.7)	1 138 (19.0)	315 (24.4)	192 (23.1)		810 (29.9)	455 (31.6)*
Current smoker, n (%)	7 015 (57.2)	3 678 (61.5)	696 (53.9)	467 (56.3)		1 471 (54.2)	703 (48.8)*
Current drinking, n (%)	7 445 (60.7)	3 543 (59.2)	768 (59.5)	507 (61.1)		1 739 (64.1)	888 (61.6)*
Diabetes mellitus, n (%)	1 308 (10.7)	408 (6.8)	132 (10.2)	113 (13.6)		404 (14.9)	251 (17.4)*
Hypertension, n (%)	4 936 (40.3)	1 761 (29.4)	545 (42.2)	337 (40.6)		1 386 (51.1)	907 (62.9)*
Low blood HDL-C, n (%)	5 676 (46.3)	2 179 (36.4)	601 (46.6)	371 (44.7)		1 559 (57.5)	966 (67.0)*
BMI, mean±SD, kg/m^2^	23.9 ± 3.4	21.1 ± 1.7	25.2 ± 1.0	22.6 ± 1.2		26.0 ± 1.1	30.2 ± 2.2*
WC, mean±SD, cm	82.6 ± 10.2	74.8 ± 5.6	80.2 ± 4.3	88.8 ± 4.1		91.0 ± 4.4	97.5 ± 7.9*
Married/cohabiting, n (%)	10 225 (83.4)	4 783 (79.9)	1 079 (83.6)	714 (86.0)		2 386 (87.9)	1 263 (87.6)*
Retired personnel, n (%)	628 (5.1)	181 (3.0)	64 (5.0)	63 (7.6)		224 (8.3)	96 (6.7)*
PA, M (P25, P75), MET-h/d	16.5 (9.4-33.2)	19.2 (10.1-37.9)	18.3 (10.1-35.6)	14.4 (8.7-27.2)		14.2 (9.0-27.2)	14.3 (9.0-27.2)*
Consuming vegetables daily, n (%)	11 552 (94.2)	5 647 (94.4)	1 213 (94.0)	786 (94.7)		2 559 (94.3)	1 347 (93.5)
Consuming fruit daily, n (%)	3 138 (25.6)	1 336 (22.8)	315 (24.4)	247 (29.8)		768 (28.3)	442 (30.7)*
**Female**							
Number	14 853 (100.0)	6 325 (42.6)	1 312 (8.8)	1 419 (9.6)		3 633 (24.5)	2 164 (14.6)
Age, M (P25, P75), y	47 (38, 57)	44 (34, 55)	44 (38, 53)	53 (41, 61)		51 (42, 59)	50 (42, 59)*
Urban residence, n (%)	5 982 (40.3)	2 496 (39.5)	521 (39.7)	544 (38.3)		1 540 (42.4)	881 (40.7)*
High school and above, n (%)	2 730 (18.4)	1 397 (22.1)	260 (19.8)	219 (15.4)		549 (15.1)	305 (14.1)*
Current smoker, n (%)	512 (3.4)	209 (3.3)	36 (2.7)	64 (4.5)		128 (3.5)	75 (3.5)*
Current drinking, n (%)	2 429 (16.4)	1 115 (17.6)	212 (16.2)	258 (18.2)		572 (15.7)	272 (12.6)*
Diabetes mellitus, n (%)	1 460 (9.8)	321 (5.1)	87 (6.6)	177 (12.5)		496 (13.7)	379 (17.5)*
Hypertension, n (%)	5 714 (38.5)	1 520 (24.0)	489 (37.3)	563 (39.7)		1 786 (49.2)	1 356 (62.7)*
Low blood HDL-C, n (%)	5 735 (38.6)	1 977 (31.3)	564 (43.0)	524 (36.9)		1 610 (44.3)	1 060 (49.0)*
BMI, mean±SD, kg/m^2^	24.2 ± 3.6	21.2 ± 1.7	25.2 ± 1.0	22.6 ± 1.3		26.0 ± 1.1	30.4 ± 2.3*
WC, mean±SD, cm	79.8 ± 9.9	71.5 ± 5.0	75.7 ± 3.6	83.8 ± 3.9		86.4 ± 5.0	92.9 ± 8.4*
Married/cohabiting, n (%)	12 480 (84.0)	5 157 (81.5)	1 155 (88.0)	1 179 (83.1)		3 123 (86.0)	1 866 (86.2)*
Retired personnel, n (%)	897 (6.0)	252 (4.0)	60 (4.6)	111 (7.8)		311 (8.6)	163 (7.5)*
PA, M (P25, P75), MET-h/d	15.1 (10.1-23.3)	15.2 (10.2-23.8)	15.2 (10.3-24.1)	13.8 (9.4-21.3)		15.0 (10.2-24.1)	14.5 (9.9-22.5)*
Consuming vegetables daily, n (%)	14 089 (94.9)	6 012 (95.1)	1 245 (94.9)	1 334 (94.0)		3 446 (94.9)	2 052 (94.8)
Consuming fruit daily, n (%)	5 454 (36.7)	2 314 (36.6)	504 (38.4)	491 (34.6)		1 329 (36.6)	816 (37.7)

*BMI* body mass index,  *WC *waist circumference,  *MET* metabolic equivalent,  *HDL-C* high-density lipoprotein cholesterol,  PA physical activity

* Comparison of differences in baseline characteristics between anthropometric groups, *P *< 0.05

### Associations of Combining BMI and WC with Stroke

In males, 597 stroke events were observed during 6.40 ± 0.56 years follow-up. After adjusting for relevant confounding factors, the normal weight or underweight/normal WC group was the reference group. Among the general male population, the risk of stroke onset was increased by 45% (HR = 1.45 95%CI: 1.07-1.97), 41% (HR = 1.41 95%CI: 1.14-1.75), and 46% (HR = 1.46 95%CI: 1.11-1.91) in the normal weight/abdominal obesity, overweight/abdominal obesity, obesity/abdominal obesity groups, respectively (see Table 2). In male with baseline hypertension, diabetes, low HDL-C, or CVD risk factors, we found that stroke risk remained increased in the normal weight or underweight/abdominal obesity, overweight/abdominal obesity, and obesity/abdominal obesity groups (see Table S1 in the Additional file 1).

**Table 2:** Associations of Combining BMI and WC with Stroke in Males

	Normal weight or underweight/ Normal WC	Overweight/ Normal WC	Normal weight or underweight/ Abdominal obesity	Overweight/ Abdominal obesity	Obesity/ Abdominal obesity
**General population**					
Number of events	249	60	57	152	79
Incidence rate (no./1000 person-years)	6.51	7.21	10.78	8.74	8.53
Hazard ratio	1.00 (reference)	1.09	1.45	1.41	1.46
95%CI, without FAR	-	0.81-1.47	1.07-1.97	1.14-1.75	1.11-1.91
95%CI, with FAR	0.87-1.15	0.84-1.42	1.11-1.91	1.20-1.67	1.15-1.84
* P*-value^*^	-	0.561	0.017^#^	0.002^#^	0.007^#^
**Subjects with CVD risk factors**					
Number of events	171	51	47	135	73
Incidence rate (no./1000 person-years)	7.62	8.47	12.56	9.46	8.76
Hazard ratio	1.00	1.10	1.56	1.40	1.40
95%CI, without FAR	-	0.79-1.54	1.10-2.20	1.10-1.79	1.04-1.88
95%CI, with FAR	0.85-1.18	0.83-1.47	1.15-2.11	1.17-1.67	1.10-1.78
* P*-value^*^		0.561	0.012^#^	0.007^#^	0.026^#^

*CI* confidence interval, *FAR* floating absolute risk,  *BMI* body mass index,  *WC* waist circumference,  *HDL-C *high-density lipoprotein cholesterol,  *CVD* cardiovascular disease

^*^P-value for fully adjusted hazard ratio (HR)

^#^ indicates *P*<0.05

In females, 736 stroke events were observed during 6.44 ± 0.43 years follow-up. After adjusting for relevant confounding factors, as for males, the normal weight or underweight/normal WC group was the reference group. Among the females, the risk of stroke onset was increased by 33% (HR = 1.33 95%CI: 1.10-1.61), 46% (HR = 1.46 95%CI: 1.17-1.81) in the overweight/abdominal obesity, obesity/abdominal obesity groups, respectively (see Table 3). In sub-populations, we still found an increased risk of stroke in the overweight/abdominal obesity, obesity/abdominal obesity groups except for the diabetes subpopulation (see Table S2 in the Additional file 1).

**Table 3:** Associations of Combining BMI and WC with Stroke in Females

	Normal weight or underweight/ Normal WC	Overweight/ Normal WC	Normal weight or underweight/ Abdominal obesity	Overweight/ Abdominal obesity	Obesity/ Abdominal obesity
**General population**					
Number of events	220	46	77	242	151
Incidence rate (no./1000 person-years)	5.40	5.42	8.43	10.37	10.87
Hazard ratio	1.00 (reference)	0.90	0.98	1.33	1.46
95%CI, without FAR	-	0.65-1.25	0.75-1.29	1.10-1.61	1.17-1.81
95%CI, with FAR	0.87-1.15	0.67-1.21	0.78-1.24	1.17-1.52	1.24-1.72
* P*-value^*^		0.533	0.901	0.004^#^	0.001^#^
**Subjects with CVD risk factors**					
Number of events	158	39	60	216	137
Incidence rate (no./1000 person-years)	7.81	6.94	10.09	12.55	11.83
Hazard ratio	1.00	0.84	0.90	1.27	1.27
95%CI, without FAR	-	0.59-1.20	0.66-1.22	1.03-1.58	1.00-1.61
95%CI, with FAR	0.85-1.18	0.61-1.16	0.69-1.17	1.11-1.51	1.07-1.51
* P*-value^*^		0.340	0.502	0.028^#^	0.046^#^

*CI* confidence interval,  * FAR* floating absolute risk,  *BMI* body mass index,  *WC* waist circumference,  *HDL-C* high-density lipoprotein cholesterol,  *CVD* cardiovascular disease

^*^P-value for fully adjusted hazard ratio (HR)

^#^ indicates *P *< 0.05

### Subgroup analysis and sensitivity analysis

**Fig. 1:**
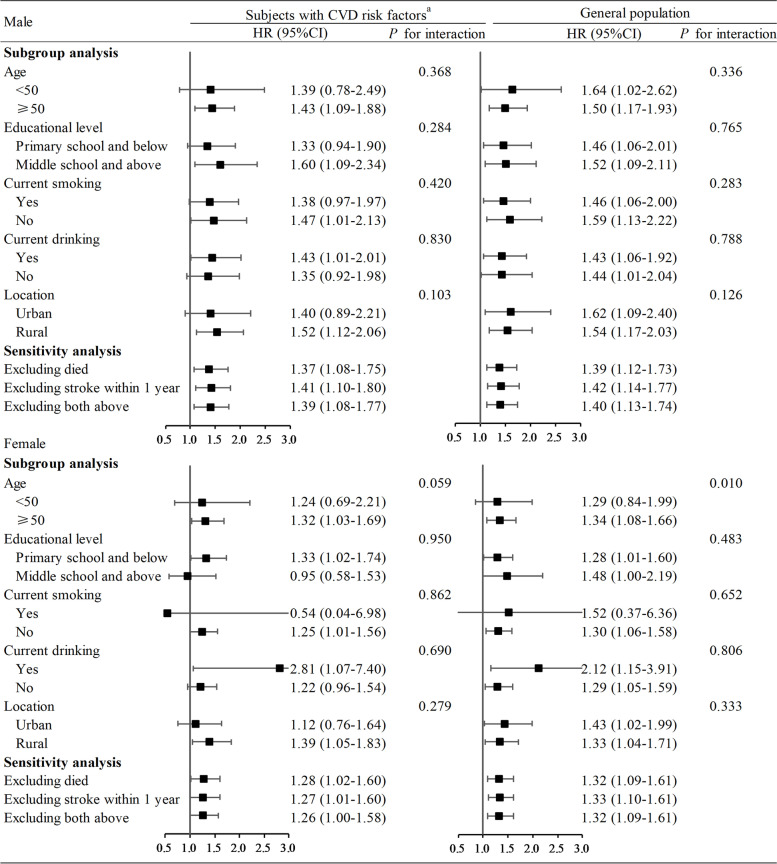
Result of Overweight/Abdominal obesity in Subgroup and Sensitivity Analysis for Incident Stroke in Males and Females. ^a^CVD risk factors denotes subjects with hypertension, diabetes, or low blood HDL-C. CVD, cardiovascular disease; HDL-C, high-density-lipoprotein cholesterol. Stratified by region, age and adjusted for educational level, marital status, location, occupational, smoking, current drinking, physical activity, self-rated health, consumption of fresh vegetables, consumption of fresh fruit, insufficient intake of vegetables and fruit, not included subgroup. The black boxes represent hazard ratios, and the horizontal lines represent 95% confidence intervals. Likelihood ratio tests were performed to examine the interaction of anthropometric groups and baseline characteristic

The subgroup analysis and sensitivity analysis results for the overweight/abdominal obesity group are summarized in Fig. 1. In the subgroup analysis we only found an interaction between age and anthropometric group (*P* for interaction = 0.010) in the general female population. Moreover, higher hazard ratio values for stroke onset were found at age ≥50 years than the age <50 years. In the sensitivity analysis, 356 (289) died and 36 (33) people diagnosed with stroke within the first year among the general male subjects (male subjects with CVD risk factors) were excluded. Furthermore, 220 (186) people died and 37 (32) people diagnosed with stroke within the first year among the general female population (female subjects with CVD risk factors) were excluded. We both found no change in the sensitivity analysis results. Results of subgroup analyses and sensitivity analyses for other anthropometric groups are presented in Additional file 1 Table S3.

## Discussion

In our study, we found not only overweight/obesity with abdominal obesity increased the risk of stroke onset, but also normal weight or underweight with abdominal obesity increased the risk of stroke in male. It suggests that males with abdominal obesity are at increased risk of stroke even at a normal BMI. A Japanese study showed a 22% (OR = 1.22 95%CI: 1.17-1.27) increased risk of hypertension, 81% (OR = 1.81 95%CI: 1.74-1.89) increased risk of dyslipidemia and 35% (OR = 1.35 95%CI: 1.25-1.46) increased risk of diabetes in normal weight abdominal obesity males (normal weight with normal WC as a reference) [36]. Moreover, some studies have shown an association between normal weight abdominal obesity and stroke risk factors such as hypertension, diabetes, and dyslipidemia [37, 38]. It may be one reason why normal-weight abdominal obesity increased the risk of stroke in males in our study. However, we may overlook this group of people (normal weight with abdominal obesity) because we tend to treat this group as a normal population, namely no overweight/obesity. Normal weight with abdominal obesity people may not receive appropriate health education, and normal weight people have lower central obesity awareness and control [36, 39]. Thus, it is important to screen normal weight with abdominal obesity using a combination of body mass index and waist circumference in males [39, 40].

In the female general population, we only found an increased risk of stroke in overweight/obesity with abdominal obesity. Previous studies have shown that normal weight abdominal obesity is associated with CVD risk factors in female [37, 38]. However, we found no increased risk of stroke in this population (normal weight or underweight abdominal obesity), which may have been influenced by other factors in our study. Specific factors need to be further explored in our future studies. A study found that elevated trunk fat and reduced leg fat increase CVD risk in women of normal weight [25]. It implies that the risk of CVD may be different in different regions of body fat deposition in a normal weight population of women. The present study suggests that it is may be more beneficial to control waist circumference rather than body mass index in overweight/obesity for stroke prevention. Therefore, it is recommended to combine body mass index and waist circumference to evaluate the obesity pattern. Previous studies also recommended combining body mass index and waist circumference to evaluate obesity patterns [11, 41].

We analyzed hypertension and non-hypertension, diabetes and non-diabetes, low HDL-C and non-low HDL-C, CVD risk factors and non-CVD risk factors to understand anthropometric groups’ impact on stroke risk in the different subpopulations. We found that in male with hypertension, diabetes, low HDL-C or CVD risk factors populations all increased risk of stroke in the normal weight or underweight with abdominal obesity and overweight/obesity with abdominal obesity population, and also found hypertension, diabetes and low HDL-C no modifying effects on the anthropometric group and stroke incidence risk. Prior studies in the prediabetic population in China shown that compared with normal BMI without central obesity, the risk of cardiovascular events was increased among men central obesity with high BMI (HR, 95%CIs were 1.32, 1.05 – 1.67 for BMI 24 – 27.9 kg/m^2^ and 1.31, 1.03 – 1.66 for BMI ≥ 28 kg/m^2^, respectively) [42], consistent with the results of our study. We also found that hypertension, diabetes, and low HDL-C have no modifying effects on the anthropometric group and stroke incidence risk. We further confirmed the need for a combination of BMI and waist circumference to assess obesity patterns.

Previous studies have confirmed that obesity defined by BMI increases risk of cardiovascular disease. However, BMI could not reflect the apportionment of body fat. The distribution of body fat is closely related to the incidence of cardiovascular diseases. Waist circumference can be used as a supplement to BMI and roughly reflect the distribution of body fat. A previous study found that both WC and BMI were better than BMI or WC alone to predict cardiovascular disease [43]. The possible mechanism linking the combination of BMI and WC and risk of stroke might be the increased oxidative stress and insulin resistance resulting from increased body fat, and accelerated atherosclerosis [44]. Moreover, some studies showed that the combination of BMI and waist circumference could better evaluate the proportion of body fat and predict visceral obesity which would allow for a more comprehensive assessment of a person’s obesity [45–47]. We conducted subgroup and sensitivity analyses in the general population and population with the CVD risk factors. We only found an effect modification of age on the anthropometric group and stroke onset in the general female population. Higher hazard values for age greater than or equal to 50 years of obesity with the abdominal obesity population. We excluded those who died and within the first year stroke with the results unchanged and confirmation of stability of study results.

Several studies also examined the relationship between the combination of body mass index and waist circumference and the risk of stroke mortality. The Third National Health and Nutrition Examination Survey (NHANES III) showed that the total risk of mortality hazard ratios 1.87 (95%CI, 1.53-2.29) in the normal weight central obesity population in men and 1.48 (95%CI, 1.35-1.62) in women, and overweight without central obesity have lower all-cause mortality among men [23, 24]. It demonstrates that the combination of body mass index and waist circumference is also significant in stroke mortality.

Our study has the strength of a prospective cohort study. Height, weight, and waist circumference were measured by trained and qualified investigators using a uniform measurement protocol. In addition, stroke outcomes were confirmed by a face-to-face survey with a structured questionnaire, and further confirmed by review of medical records (e.g., clinical diagnosis information, imaging scans) and checking with Cardiovascular Event Reporting System. Previous studies have shown that strokes can also be reasonably assessed using self-administered questionnaires [48]. We also calculated the hazard ratio (HR) (with the same hazard ratio value as the traditional method) and 95% confidence interval of HR using a floating absolute risk (FAR) method [49].

However, our study has limitations. First, there are some loss-of-follow up. Although loss of follow-up rate was less than 30%, the loss of follow-up may have biased the results (e.g., over or underestimation of the results). Second, body mass index and waist circumference changes were not be measured after the baseline measurements as fluctuations in body weight and waist circumference may affect the risk of stroke. Third, we did not further analyze the stroke subtypes due to sample size. It requires us to expand our populations to study the situation of stroke subtypes in the future. Fourth, abdominal obesity is assessed only using waist circumference indicator, and several studies have shown that waist-to-height ratio may be better than waist circumference [50, 51]. Moreover, it has been noted that the correlation of BMI, waist circumference and waist to height ratio are up to 0.9, making it difficult to disentangle these parameters [52]. One suggestion, has been to use an index such as ABSI, that normalizes waist circumference to BMI [53, 54]. Fifth, some confounding factors are not included (e.g., air pollution). Finally, the results are possible limited due to the Chinese population.

## Conclusions

Compared with the normal weight or underweight with normal WC group, overweight/obesity with abdominal obesity or normal BMI with abdominal obesity increase the risk of incident stroke in males, and overweight/obesity with abdominal obesity increases the risk of incident stroke in females. Body mass index and waist circumference should be combined to assess obesity patterns, and identify individuals most in need of weight control to prevent stroke.

## Supplementary Information

**Additional file 1:** **Figure S1.** Flowchart of participant inclusion. **Figure S2.** Prevalence (95%CI) of General Obesity by Sex in General Population and Sub-populations. **Figure S3.** Prevalence (95%CI) of Abdominal Obesity by Sex in General Population and Sub-populations. **TableS1.** Associations of Combining BMI and WC with Stroke in Males Subpopulation. **TableS2.** Associations of Combining BMI and WC with Stroke in Females Subpopulation. **TableS3.** Subgroup analysis and Sensitivity analysis for incident stroke in general and with CVD risk factor population in male and female (not included results of Overweight/High WC). 

## Data Availability

The datasets used and analysed during the current study are available from the corresponding author on reasonable request.

## Abbreviations

BMI: Body Mass Index
WC: Waist circumference
CCDRFS: China Chronic Disease Risk Factor Surveillance
FAR: Floating Absolute Risk
