# Supplementary material for: Combined consideration of body mass index and waist circumference identifies obesity patterns associated with risk of stroke in a Chinese prospective cohort study
Source: BMC Public Health. 2022 Feb 18;22:347. doi: 10.1186/s12889-022-12756-2 (PMC8855545; doi:10.1186/s12889-022-12756-2)
Supplement: Supplementary file 1 — Additional file 1: Figure S1. Flowchart of participant inclusion. Figure S2. Prevalence (95%CI) of General Obesity by Sex in General Population and Sub-populations. Figure S3. Prevalence (95%CI) of Abdominal Obesity by Sex in General Population and Sub-populations. TableS1. Associations of Combining BMI and WC with Stroke in Males Subpopulation. TableS2. Associations of Combining BMI and WC with Stroke in Females Subpopulation. TableS3. Subgroup analysis and Sensitivity analysis for incident stroke in general and with CVD risk factor population in male and female (not included results of Overweight/High WC). [file 12889_2022_12756_MOESM1_ESM.docx]

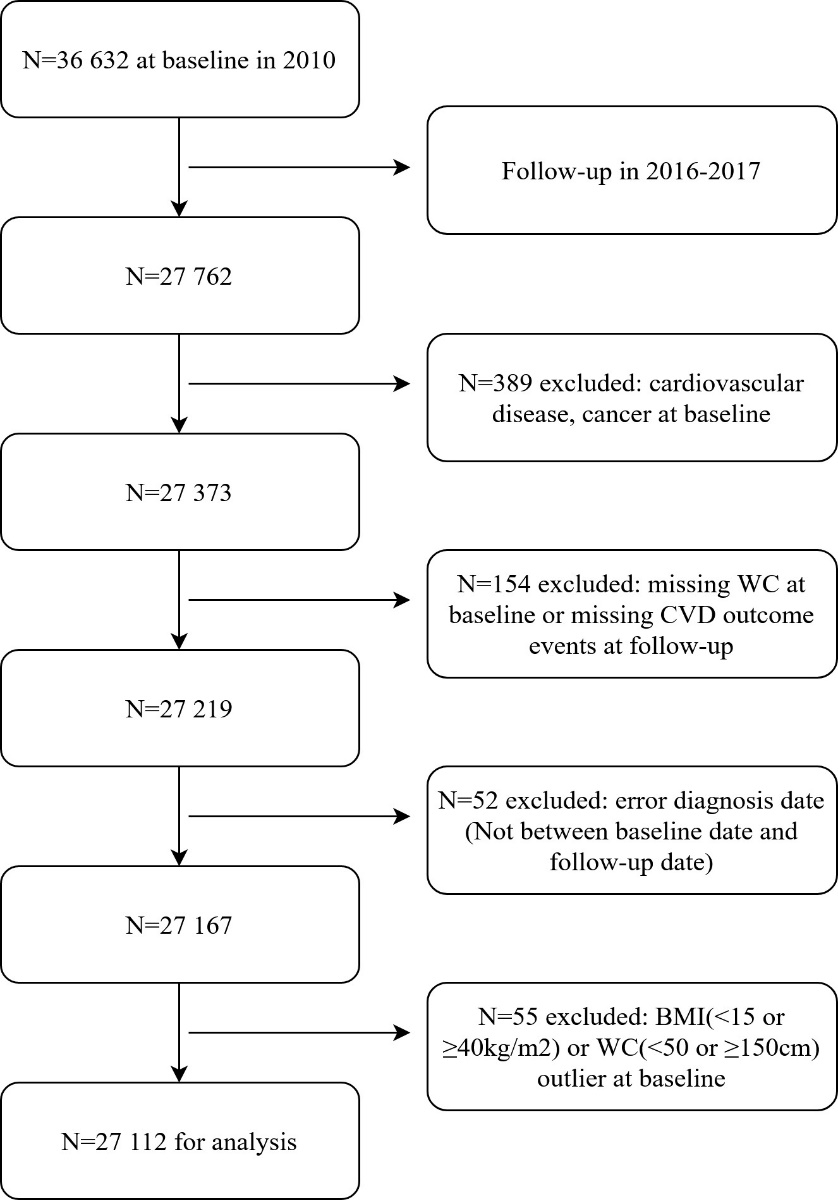


**Figure .S1** Flowchart of participant inclusion.


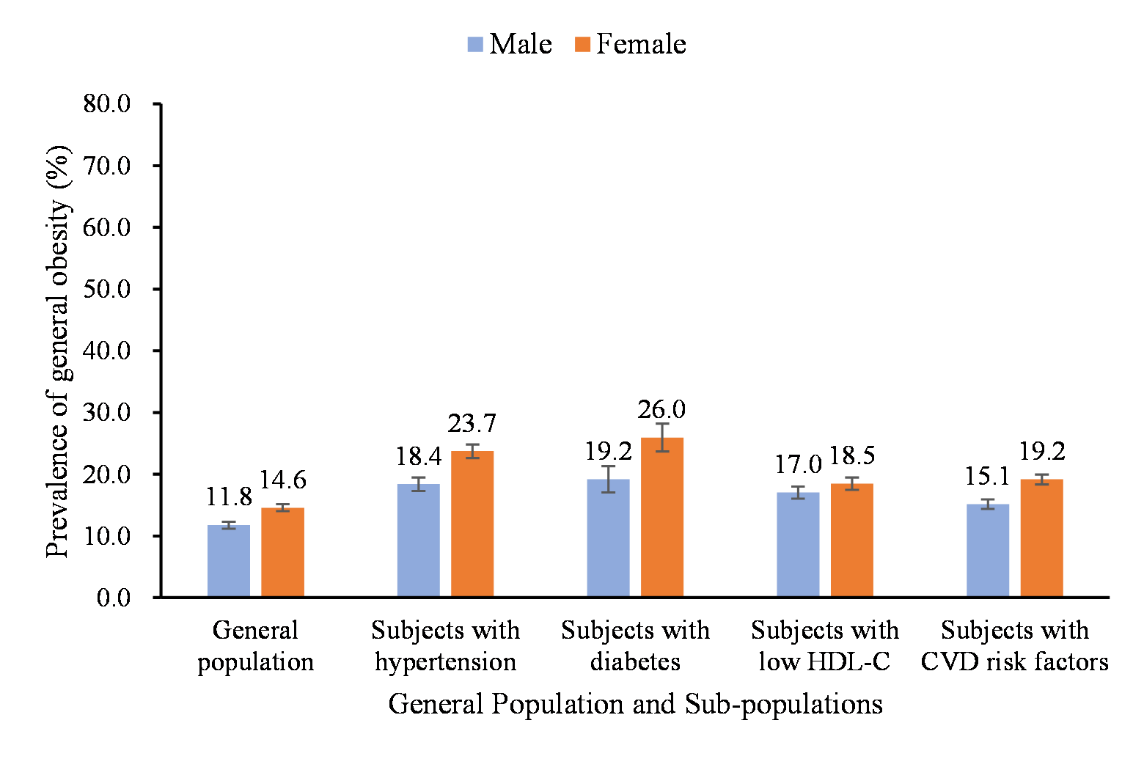


**Figure. S2** **Prevalence (95%CI) of General Obesity by Sex in General Population and Sub-populations**


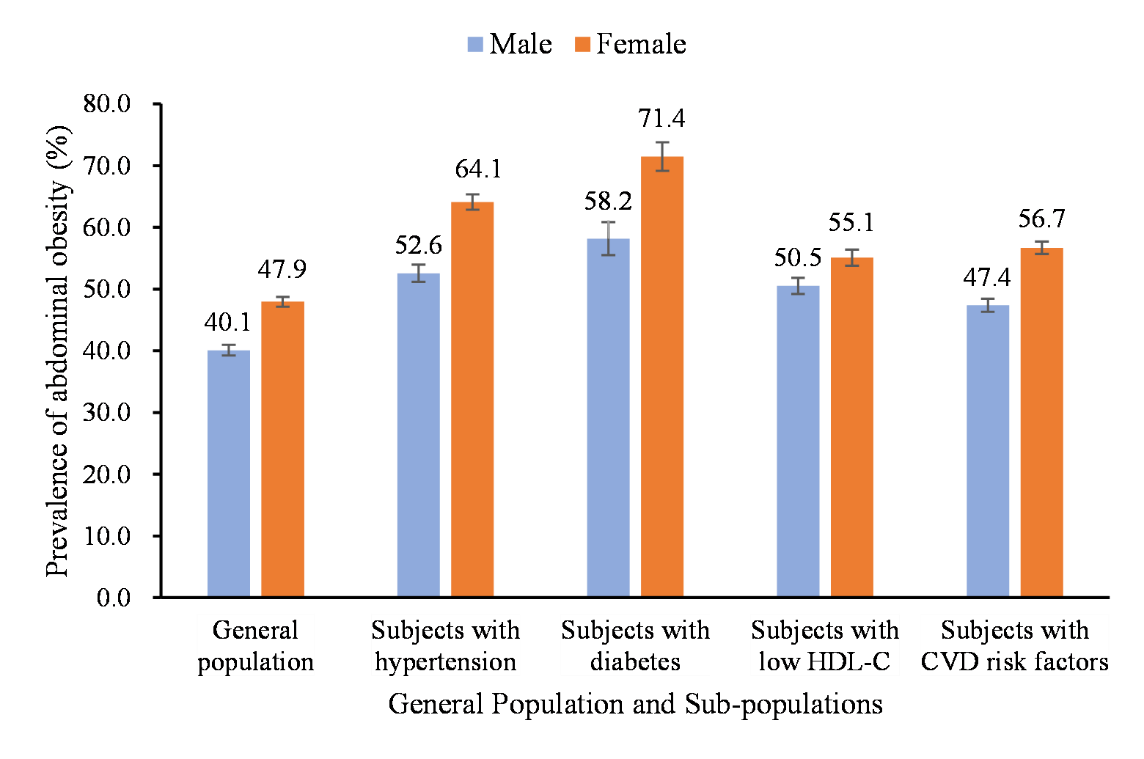


**Figure. S3 Prevalence (95%CI) of Abdominal Obesity by Sex in General Population and Sub-populations**

**Table S1** **Associations of Combining BMI and WC with Stroke in Males Subpopulation**

|  | Normal weight or underweight/  Normal WC | Overweight/  Normal WC | Normal weight or underweight/  Abdominal obesity | Overweight/  Abdominal obesity | Obesity/  Abdominal obesity |
| --- | --- | --- | --- | --- | --- |
| **Subjects with hypertension** |  |  |  |  |  |
| Number of events | 130 | 40 | 35 | 117 | 61 |
| Incidence rate (no./1000 person-years) | 11.72 | 11.45 | 16.45 | 13.24 | 10.52 |
| Hazard ratio | 1.00 (reference) | 1.03 | 1.51 | 1.52 | 1.40 |
| 95%CI, without FAR | - | 0.72-1.48 | 1.03-2.21 | 1.17-1.98 | 1.01-1.94 |
| 95%CI, with FAR | 0.83-1.21 | 0.75-1.41 | 1.08-2.11 | 1.27-1.83 | 1.08-1.81 |
| *P*-value^*^ |  | 0.865 | 0.035^#^ | 0.002^#^ | 0.046^#^ |
| **Subjects with diabetes** |  |  |  |  |  |
| Number of events | 20 | 8 | 14 | 27 | 14 |
| Incidence rate (no./1000 person-years) | 7.85 | 9.41 | 19.42 | 10.48 | 8.74 |
| Hazard ratio | 1.00 (reference) | 1.62 | 2.91 | 2.59 | 2.73 |
| 95%CI, without FAR | - | 0.55-4.74 | 1.14-7.38 | 1.14-5.86 | 1.03-7.25 |
| 95%CI, with FAR | 0.53-1.88 | 0.65-4.03 | 1.38-6.10 | 1.58-4.25 | 1.36-5.51 |
| *P*-value^*^ |  | 0.380 | 0.025^#^ | 0.022^#^ | 0.043^#^ |
| **Subjects with low HDL-C** |  |  |  |  |  |
| Number of events | 75 | 31 | 22 | 76 | 50 |
| Incidence rate (no./1000 person-years) | 5.36 | 7.99 | 9.28 | 7.59 | 8.04 |
| Hazard ratio | 1.00 (reference) | 1.55 | 1.71 | 1.62 | 1.60 |
| 95%CI, without FAR | - | 0.96-2.49 | 0.95-3.06 | 1.11-2.36 | 1.05-2.45 |
| 95%CI, with FAR | 0.76-1.32 | 1.04-2.30 | 1.02-2.85 | 1.25-2.10 | 1.16-2.21 |
| *P*-value^*^ |  | 0.073 | 0.073 | 0.013^#^ | 0.030^#^ |

CI, confidence interval; FAR, floating absolute risk; BMI, body mass index; WC, waist circumference; HDL-C, high-density lipoprotein cholesterol; CVD, cardiovascular disease.

^*^*P*-value for fully adjusted hazard ratio (HR).

^#^ indicates *P*<0.05.

**Table S2 Associations of Combining BMI and WC with Stroke in Females Subpopulation**

|  | Normal weight or underweight/  Normal WC | Overweight/  Normal WC | Normal weight or underweight/  Abdominal obesity | Overweight/  Abdominal obesity | Obesity/  Abdominal obesity |
| --- | --- | --- | --- | --- | --- |
| **Subjects with hypertension** |  |  |  |  |  |
| Number of events | 118 | 28 | 47 | 189 | 125 |
| Incidence rate (no./1000 person-years) | 12.19 | 8.93 | 13.02 | 16.54 | 14.40 |
| Hazard ratio | 1.00 (reference) | 0.81 | 0.90 | 1.32 | 1.25 |
| 95%CI, without FAR | - | 0.53-1.22 | 0.64-1.27 | 1.04-1.67 | 0.97-1.62 |
| 95%CI, with FAR | 0.83-1.20 | 0.55-1.17 | 0.67-1.20 | 1.14-1.52 | 1.05-1.50 |
| *P*-value^*^ |  | 0.309 | 0.544 | 0.021^#^ | 0.086 |
| **Subjects with diabetes** |  |  |  |  |  |
| Number of events | 30 | 8 | 10 | 59 | 41 |
| Incidence rate (no./1000 person-years) | 14.66 | 14.24 | 8.77 | 18.63 | 16.97 |
| Hazard ratio | 1.00 (reference) | 0.89 | 0.47 | 1.03 | 1.32 |
| 95%CI, without FAR | - | 0.35-2.25 | 0.21-1.06 | 0.59-1.80 | 0.74-2.35 |
| 95%CI, with FAR | 0.64-1.57 | 0.39-2.01 | 0.23-0.94 | 0.74-1.43 | 0.92-1.90 |
| *P*-value^*^ |  | 0.801 | 0.068 | 0.923 | 0.343 |
| **Subjects with low HDL-C** |  |  |  |  |  |
| Number of events | 71 | 23 | 25 | 111 | 74 |
| Incidence rate (no./1000 person-years) | 5.55 | 6.28 | 7.40 | 10.71 | 10.84 |
| Hazard ratio | 1.00 (reference) | 0.93 | 0.87 | 1.47 | 1.48 |
| 95%CI, without FAR | - | 0.56-1.54 | 0.53-1.42 | 1.06-2.05 | 1.03-2.12 |
| 95%CI, with FAR | 0.77-1.30 | 0.60-1.44 | 0.57-1.32 | 1.20-1.80 | 1.16-1.89 |
| *P*-value^*^ |  | 0.772 | 0.565 | 0.023^#^ | 0.033^#^ |

CI, confidence interval; FAR, floating absolute risk; BMI, body mass index; WC, waist circumference; HDL-C, high-density lipoprotein cholesterol; CVD, cardiovascular disease.

^*^*P*-value for fully adjusted hazard ratio (HR).

^#^ indicates *P*<0.05.

**Table S3** Subgroup analysis and Sensitivity analysis for incident stroke in general and with CVD risk factor population in male and female (not included results of Overweight/High WC)

| **Male** | Population with CVD risk factor | | | |  | General population | | | |  |  |  |  |  |  |  |  |  |
| --- | --- | --- | --- | --- | --- | --- | --- | --- | --- | --- | --- | --- | --- | --- | --- | --- | --- | --- |
|  | Normal BMI or underweight/ | Overweight/ | Normal BMI or underweight/ | Obesity/ |  | Normal BMI or underweight/ | Overweight/ | Normal BMI or underweight/ | Obesity/ |  |  |  |  |  |  |  |  |  |
|  | Normal WC | Normal WC | Abdominal obesity | Abdominal obesity |  | Normal WC | Normal WC | Abdominal obesity | Abdominal obesity |  |  |  |  |  |  |  |  |  |
| Subgroup analysis |  |  |  |  |  |  |  |  |  |  |  |  |  |  |  |  |  |  |
| Age |  |  |  |  |  |  |  |  |  |  |  |  |  |  |  |  |  |  |
| <50 | 1.00(reference) | 1.28(0.60-2.73) | 1.61(0.68-3.80) | 1.75(0.92-3.35) |  | 1.00(reference) | 0.99(0.51-1.93) | **2.30(1.25-4.23)** | **2.06(1.21-3.51)** |  |  |  |  |  |  |  |  |  |
| ≥50 | 1.00(reference) | 1.07(0.74-1.55) | **1.56(1.06-2.29)** | 1.41(1.00-1.98) |  | 1.00(reference) | 1.15(0.82-1.60) | 1.36(0.96-1.93) | **1.50(1.08-2.08)** |  |  |  |  |  |  |  |  |  |
| Educational level |  |  |  |  |  |  |  |  |  |  |  |  |  |  |  |  |  |  |
| Primary school and below | 1.00(reference) | 1.27(0.80-2.02) | **1.72(1.05-2.80)** | 1.15(0.71-1.88) |  | 1.00(reference) | 1.29(0.85-1.95) | 1.47(0.95-2.28) | 1.24(0.79-1.97) |  |  |  |  |  |  |  |  |  |
| middle school and above | 1.00(reference) | 1.03(0.62-1.72) | 1.38(0.79-2.40) | **1.78(1.16-2.74)** |  | 1.00(reference) | 1.08(0.69-1.68) | 1.56(0.99-2.47) | **1.87(1.27-2.74)** |  |  |  |  |  |  |  |  |  |
| Current smoking |  |  |  |  |  |  |  |  |  |  |  |  |  |  |  |  |  |  |
| yes | 1.00(reference) | 1.25(0.79-1.95) | **2.01(1.25-3.24)** | 1.14(0.69-1.90) |  | 1.00(reference) | 1.28(0.86-1.90) | **1.96(1.32-2.92)** | 1.31(0.83-2.07) |  |  |  |  |  |  |  |  |  |
| no | 1.00(reference) | 1.01(0.60-1.70) | 1.26(0.71-2.24) | **1.57(1.04-2.38)** |  | 1.00(reference) | 1.08(0.67-1.72) | 1.16(0.69-1.96) | **1.74(1.18-2.57)** |  |  |  |  |  |  |  |  |  |
| Current drinking |  |  |  |  |  |  |  |  |  |  |  |  |  |  |  |  |  |  |
| yes | 1.00(reference) | 0.83(0.50-1.36) | **2.20(1.40-3.45)** | **1.60(1.05-2.46)** |  | 1.00(reference) | 0.93(0.60-1.42) | **1.92(1.31-2.81)** | **1.66(1.13-2.43)** |  |  |  |  |  |  |  |  |  |
| no | 1.00(reference) | 1.35(0.92-1.98) | 0.97(0.52-1.81) | 1.13(0.71-1.81) |  | 1.00(reference) | 1.42(0.92-2.19) | 1.04(0.59-1.84) | 1.34(0.86-2.09) |  |  |  |  |  |  |  |  |  |
| Location |  |  |  |  |  |  |  |  |  |  |  |  |  |  |  |  |  |  |
| urban | 1.00(reference) | 0.68(0.32-1.43) | 1.36(0.68-2.74) | 1.45(0.83-2.54) |  | 1.00(reference) | 0.76(0.41-1.44) | 1.33(0.71-2.49) | **1.77(1.07-2.92)** |  |  |  |  |  |  |  |  |  |
| rural | 1.00(reference) | 1.24(0.84-1.83) | **1.75(1.14-2.69)** | 1.44(0.99-2.10) |  | 1.00(reference) | 1.29(0.91-1.83) | **1.58(1.09-2.30)** | **1.60(1.13-2.27)** |  |  |  |  |  |  |  |  |  |
| Sensitivity analysis |  |  |  |  |  |  |  |  |  |  |  |  |  |  |  |  |  |  |
| Excluding died | 1.00(reference) | 1.11(0.80-1.55) | **1.57(1.11-2.22)** | **1.45(1.07-1.96)** |  | 1.00(reference) | 1.10(0.81-1.48) | **1.46(1.08-1.98)** | **1.53(1.16-2.02)** |  |  |  |  |  |  |  |  |  |
| Excluding stroke within 1 year | 1.00(reference) | 1.07(0.76-1.50) | **1.51(1.06-2.16)** | **1.50(1.10-2.04)** |  | 1.00(reference) | 1.07(0.79-1.44) | **1.42(1.04-1.94)** | **1.58(1.20-2.10)** |  |  |  |  |  |  |  |  |  |
| Excluding both above | 1.00(reference) | 1.08(0.77-1.52) | **1.51(1.06-2.16)** | **1.49(1.09-2.02)** |  | 1.00(reference) | 1.07(0.79-1.44) | **1.41(1.03-1.93)** | **1.59(1.20-2.10)** |  |  |  |  |  |  |  |  |  |
| **Female** | | | | | | | | | |  |  |  |  |  |  |  |  |  |
| Subgroup analysis |  |  |  |  |  |  |  |  |  |  |  |  |  |  |  |  |  |  |
| Age |  |  |  |  |  |  |  |  |  |  |  |  |  |  |  |  |  |  |
| <50 | 1.00(reference) | 0.90(0.41-1.98) | 0.36(0.10-1.29) | 1.52(0.82-2.83) |  | 1.00(reference) | 0.82(0.44-1.54) | 0.58(0.24-1.36) | **1.79(1.13-2.84)** |  |  |  |  |  |  |  |  |  |
| ≥50 | 1.00(reference) | 0.88(0.57-1.36) | 1.03(0.73-1.45) | 1.30(0.99-1.72) |  | 1.00(reference) | 0.94(0.64-1.38) | 1.05(0.79-1.40) | **1.38(1.08-1.78)** |  |  |  |  |  |  |  |  |  |
| Educational level |  |  |  |  |  |  |  |  |  |  |  |  |  |  |  |  |  |  |
| Primary school and below | 1.00(reference) | 0.97(0.61-1.54) | 1.02(0.70-1.48) | 1.30(0.96-1.77) |  | 1.00(reference) | 0.94(0.63-1.42) | 0.98(0.72-1.34) | **1.34(1.03-1.75)** |  |  |  |  |  |  |  |  |  |
| middle school and above | 1.00(reference) | 0.64(0.32-1.29) | 0.73(0.35-1.52) | 1.25(0.75-2.08) |  | 1.00(reference) | 0.85(0.47-1.53) | 1.03(0.57-1.87) | **1.82(1.18-2.81)** |  |  |  |  |  |  |  |  |  |
| Current smoking |  |  |  |  |  |  |  |  |  |  |  |  |  |  |  |  |  |  |
| yes | 1.00(reference) | 0.12(0.00-8.99) | 0.35(0.02-7.13) | 0.41(0.03-4.92) |  | 1.00(reference) | 0.82(0.08-8.67) | 1.21(0.19-7.57) | 0.77(0.16-3.63) |  |  |  |  |  |  |  |  |  |
| no | 1.00(reference) | 0.86(0.60-1.25) | 0.89(0.65-1.22) | **1.30(1.02-1.66)** |  | 1.00(reference) | 0.93(0.66-1.30) | 0.96(0.73-1.28) | **1.46(1.17-1.83)** |  |  |  |  |  |  |  |  |  |
| Current drinking |  |  |  |  |  |  |  |  |  |  |  |  |  |  |  |  |  |  |
| yes | 1.00(reference) | 0.23(0.01-3.63) | 3.94(1.21-12.86) | 1.67(0.57-4.88) |  | 1.00(reference) | 0.58(0.19-1.80) | 1.84(0.79-4.27) | 1.43(0.64-3.19) |  |  |  |  |  |  |  |  |  |
| no | 1.00(reference) | 0.91(0.62-1.33) | 0.80(0.57-1.14) | **1.32(1.02-1.70)** |  | 1.00(reference) | 0.94(0.66-1.34) | 0.97(0.72-1.31) | **1.53(1.21-1.93)** |  |  |  |  |  |  |  |  |  |
| Location |  |  |  |  |  |  |  |  |  |  |  |  |  |  |  |  |  |  |
| urban | 1.00(reference) | 0.59(0.29-1.17) | 1.03(0.60-1.78) | 1.20(0.79-1.84) |  | 1.00(reference) | 0.69(0.37-1.28) | 1.22(0.76-1.94) | **1.51(1.03-2.21)** |  |  |  |  |  |  |  |  |  |
| rural | 1.00(reference) | 0.99(0.63-1.56) | 0.84(0.56-1.26) | 1.34(0.98-1.85) |  | 1.00(reference) | 1.00(0.67-1.51) | 0.91(0.64-1.30) | **1.46(1.10-1.94)** |  |  |  |  |  |  |  |  |  |
| Sensitivity analysis |  |  |  |  |  |  |  |  |  |  |  |  |  |  |  |  |  |  |
| Excluding died | 1.00(reference) | 0.89(0.61-1.30) | 0.92(0.66-1.27) | **1.30(1.01-1.67)** |  | 1.00(reference) | 0.90(0.65-1.25) | 0.98(0.75-1.29) | **1.44(1.16-1.80)** |  |  |  |  |  |  |  |  |  |
| Excluding stroke within 1 year | 1.00(reference) | 0.89(0.61-1.30) | 0.93(0.66-1.30) | **1.30(1.01-1.68)** |  | 1.00(reference) | 0.91(0.65-1.27) | 0.98(0.74-1.29) | **1.43(1.14-1.79)** |  |  |  |  |  |  |  |  |  |
| Excluding both above | 1.00(reference) | 0.90(0.61-1.31) | 0.92(0.66-1.28) | **1.30(1.00-1.68)** |  | 1.00(reference) | 0.92(0.66-1.28) | 0.98(0.74-1.29) | **1.43(1.14-1.79)** |  |  |  |  |  |  |  |  |  |

CVD, cardiovascular disease; BMI, body mass index; WC, waist circumference; HDL-C, high-density-lipoprotein cholesterol.

Stratified by region, age and adjusted for educational level, marital status, location, occupational, smoking, current drinking, physical activity, self-rated health, consumption of fresh vegetables, consumption of fresh fruit, insufficient intake of vegetables and fruit.

^b^ population with hypertension, diabetes, low blood HDL-C.

Bold if P<0.05.
